# Initial assessment of treatment of talar posterior process fractures with open reduction and percutaneous fixation

**DOI:** 10.1038/s41598-020-77151-6

**Published:** 2020-11-19

**Authors:** Haijiao Mao, Haiqing Wang, Jiyuan Zhao, Linger Wang, Liwei Yao, Ke Wei

**Affiliations:** 1 Department of Orthopaedic Surgery, The Affiliated Hospital of Medical School, Ningbo University, No. 247, Renming Road, Jiangbei District, Ningbo, Zhejiang China; 2 Department of Orthopaedic Surgery, Ningbo No. 6 Hospital, Ningbo, Zhejiang China; 3 The Medical School of Ningbo University, Ningbo, Zhejiang China; 4 Department of Orthopaedic Surgery, Ningbo No. 9 Hospital, Ningbo, Zhejiang China

**Keywords:** Anatomy, Musculoskeletal system, Health care, Fracture repair

## Abstract

The purpose of this study was to provide an initial assessment of treatment for talar posterior process fractures using open reduction and internal fixation (ORIF) through posteromedial approach and percutaneous screw fixation. From January 2014 to December 2018, 12 cases with displaced fracture of talar posterior process were treated in our department. The clinical and radiological results were assessed after 4 and 12 months of operation with Visual Analog Scale (VAS) pain and American Orthopedic Foot and Ankle Society (AOFAS) scores. ORIF was performed in four of the cases and percutaneous screw fixation was performed in eight of the cases. The average follow-up period was 13 months. Complications such as wound infection, nerve injury, screw loosening, malunion or nonunion of fracture were absent. For clinical assessment, considerable mprovements were observed for the AOFAS and VAS scores at 4 and 12 months postoperatively for both techniques. There was no significant difference for AOFAS scores and VAS scores between the two techniques (*p* > 0.05). Both techniques showed good functional outcome and were performed for posterior talar process fracture following the fracture displacement guidelines. Percutaneous screw fixation treatment with computer-assisted three-dimensional evaluation shortened the operation time and reduced incidences of surgical complications.

## Introduction

Fractures of talar posterior process are very uncommon injuries as these fractures cannot be discovered with standard X-ray^1,2^. This type of fracture may result in severe complications due to missed diagnosis, delayed or inappropriate treatment. It is necessary to avoid complications of pain, nonunion and tarsal tunnel syndrome for the treatment of these fractures^3–5^. The talar posterior process consists of the lateral and medial tubercles, which serve as attachments for the posterior talofibular and talotibial ligaments, respectively. The groove for the flexor hallucis longus tendon between the two tubercles is located in the middle. Fractures of talar posterior process involve an isolated fracture of either medial or lateral tubercle and simultaneous fracture of the medial and lateral tubercles (entire posterior process fractures)^4,6^.

Most reports of talar posterior process fractures are associated with subtalar dislocation^7–9^ and the recommended treatment is early open reduction and internal fixation (ORIF) or percutaneous screw fixation^8^. Subtalar dislocation accounts for 1–2% of all dislocations which makes treatment methods of talar posterior process fracture extremely rare^10^. ORIF for extensive posterior talar fractures has been reported to provide good functional outcome, quality of life and patient satisfaction^11^. Therefore, this technique is the most common treatment described in previous literature^12–14^.

ORIF is a complicated technique due to the complex anatomical structure close to the ankle joint, subtalar joint, neurovascular bundle and flexor hallucis longus tendon. Previously, we described a case and defined a safe zone for percutaneous screw fixation^8,15^. Previous reports have described that ORIF treatment leads to a different outcome^9,16^. A minimally invasive approach for this fracture was described, which is an attractive alternative compared to major open operation. This approach is especially suitable for patients who have multiple comorbidities and a higher perioperative risk. Subsequently, the best surgical approach is still in controversy. Choice of optimal treatment methods depends on fragment size, degree of displacement, subtalar joint involvement and articular surface intervention.

To the author’s knowledge, there is no literature on which treatment is a better option between ORIF and percutaneous screw for posterior talar process fracture. The purpose of this study was to provide an initial assessment of treatment for talar posterior process fractures using ORIF through posteromedial approach and percutaneous screw fixation. And this study stressed early surgical intervention in displaced fracture of the talar posterior process fracture.

## Results

Twelve Asian patients with talar posterior process fracture were treated with two techniques and followed up in this study. ORIF was performed in four of the cases and percutaneous screw fixation for posterior talar fracture was performed in eight of the cases. The average follow-up period was 13 months (8–16 months). No infection or nerve injury was observed in both groups. The mean union time was 3 months (2–5 months). All cases showed no screw loosening, implant breakage, nonunion, or malunion. No patients complained of stiffness in the ankle and subtalar joint.

For clinical assessment, considerable improvements were observed for the AOFAS and VAS scores at 4 and 12 months postoperatively in both techniques. The AOFAS scores using the ORIF technique were improved from 64.5 to 83.5 at 4 months postoperatively, and to 82.8 at 12 months postoperatively. The scores using the percutaneous screw technique were improved from 63.5 to 84.1 at 4 months postoperatively and to 83.9 at 12 months postoperatively (Table 1). Excellent and good results were achieved in eight (66.7%) patients, fair results were achieved in three (25%), and poor results were observed (8.3%) for the last follow-up.

**Table 1:** Comparison AOFAS scores (Group1 = 4; Group = 8).

AOFAS scores	Group 1	Group 2	*Z*	*p*
Preoperative	64.5 ± 1.73	63.5 ± 2.07	0.774	> 0.05
4 months postoperatively	83. 5 ± 1.00	84.1 ± 1.80	0.364	> 0.05
12 months postoperatively	82.3 ± 0.96	83.9 ± 1.24	1.058	> 0.05

The *p*-values were determined using Mann Whitney U test. Significance levels are one symbol: *p* < 0.05.

Non-significant difference between preoperative AOFAS scores in group 1 and group 2.

Non-significant difference of AOFAS scores group 1 and group 2 after 4 months.

Non-significant difference of AOFAS scores in group 1 and group 2 after 12 months.

The VAS scores decreased from 4.5 preoperatively to 2.0 and 1.8 at 4 and 12 months post-operation, respectively, using the ORIF technique and from 5.3 to 2.2 and 1.5 at 4 and 12 months post-operation, respectively, using the percutaneous screw technique (Table 2). There were no significant differences for AOFAS scores and VAS scores between the two groups at 4 and 12 months postoperatively (p > 0.05) (Tables 1,2). A significant difference (p < 0.05) was detected between AOFAS and VAS scores in the two groups preoperatively and postoperatively (Tables 3, 4).

**Table 2:** Comparison VAS scores (Group1 = 4; female = 8).

VAS scores	Group 1	Group 2	Z	*p*
Preoperative	4.5 ± 1.00	5.25 ± 0.88	0.573	> 0.05
4 months postoperatively	2.0 ± 0.82	2.3 ± 0.46	0.612	> 0.05
12 months postoperatively	1.8 ± 0.50	1.5 ± 0.53	0.793	> 0.05

The p-values were determined using independent sample t-test. Significance levels are one symbol: p < 0.05.

Non-significant difference between preoperative VAS scores e in group 1 and group 2.

Non-significant difference of VAS scores in group 1 and group 2 after 4 months.

Non-significant difference of VAS scores in group 1 and group 2 after 12 months.

**Table 3:** Comparison different time (Group1) (n = 4).

	AOFAS	VAS
Preoperative	64.5 ± 1.73	4.5 ± 1.00
4 months postoperatively	83. 5 ± 1.00	2.0 ± 0.82
12 months postoperatively	82.3 ± 0.96	1.8 ± 0.50
F	282.59	14.478
*p*	< 0.05	< 0.05
*p*^a^	< 0.05	< 0.05
*p*^b^	< 0.05	< 0.05
*p*^c^	0.429	0.669

*p*^a^ compare the results between preoperative with 4 months postoperatively .

*p*^b^ compare the results between preoperative with 12 months postoperatively.

*p*^c^ compare the results between 4 months with 12 months postoperatively.

**Table 4:** Comparison different time (Group 2) (n = 8).

	AOFAS	VAS
Preoperative	63.5 ± 2.07	5.25 ± 0.88
4 months postoperatively	84.1 ± 1.80	2.3 ± 0.46
12 months postoperatively	83.9 ± 1.24	1.5 ± 0.53
F	369.202	73.50
*p*	< 0.05	< 0.05
*p*^a^	< 0.05	< 0.05
*p*^b^	< 0.05	< 0.05
*p*^c^	0.777	0.032

*p*^a^ compare the results between preoperative with 4 months postoperatively.

*p*^b^ compare the results between preoperative with 12 months postoperatively.

*p*^c^ compare the results between 4 months with 12 months postoperatively.

## Discussion

Fractures of talar posterior process have three types, namely an isolated fracture of either medial or lateral tubercle and a simultaneous fracture of medial and lateral tubercles (i.e. entire posterior process fractures). The Cedell’s fracture was first described as a medial tubercle fracture due to talotibial ligament avulsion. The mechanism of injury may result from a posteromedial facet impaction^17^. Access and reduction of displaced or comminuted fracture fragments is a challenging procedure for surgeons due to the rarity of fractures of entire posterior process. There are only a few reports about cases of entire posterior process fractures, according to previous literatures^6,18^. To the author’s knowledge, there are few descriptions of surgical techniques for fractures of posterior talar process in the literature^8,13,19^. Some studies reported different types of posterior process fractures, however, none of them defined an optimized approach and treatment.

Fractures of talar posterior processes are prone to be misdiagnosed with primary plain radiographs (i.e. anteroposterior, mortise and lateral views). A previous study claimed that two oblique views at 45° and 70° of external rotation may be helpful, if the plain radiographs are unclear^20^. Some studies in the literature have reported that up to 40% of these fractures may be missed on initial presentation using X-ray ^21–23^. CT scan provides more accuracy for assessment of size, displacement, and comminution fragment of posterior talar process fracture or lateral process of the talus than plain radiographs^24^. If a fracture was suspected with plain radiographs, then an urgent CT scan was recommended in order to identify the fracture and assess the size and fracture fragment.

All cases in this study were diagnosed based on CT scans, in which the fracture line and displacement were assessed for surgical protocol design. Entire posterior process fractures are easier to identify than isolated medial or lateral tubercle fractures on plain radiographs. Even a minimal displacement of the fracture fragment may result in substantial joint misalignment and posttraumatic arthritis, posterior impingement and entrapment of the flexor hallucis longus tendon due to tibiotalar and talocalcaneal joints. Therefore, most authors in the literature consider that if the displacement of the fracture fragment is larger than 2 mm and subtalar joint surface is involved, then an urgent open reduction and internal fixation is recommended^9,16^.

The posterior talar process fracture is an uncommon fracture and is frequently undiagnosed by orthopaedic surgeons. There are few studies about this injury in the literature and most of them are case reports^6,18,25^. Subsequently, the effectiveness of the surgical techniques and approaches for posterior process fractures is still controversial. The posterolateral or posteromedial approach was performed with ORIF for posterior process fractures, according to the location of the displaced fragments.

The posterolateral approach involves a longitudinal incision between the lateral border of the fibula and the Achilles tendon^26^. The posteromedial approach is a popular incision, used by the majority of surgeons. The incision is located at the middle line between the Achilles tendon and the medial border of the medial malleolus^19^. Thus, adequate access to the fracture fragments and using lag screws for fixation is achieved and for the neurovascular bundle to remain unharmed. The minimally invasive percutaneous screw fixation technique was described by few reports^8,15,27,28^. Treatment options for the talar neck fracture and medial and lateral tubercle fracture of posterior talar process were described in most reports. To the authors’ knowledge, no study in the literature described in detail the initial assessment of ORIF and percutaneous screw fixation treatments.

Initial assessment of short-term results in patients who underwent internal fixation using ORIF and percutaneous screw techniques for posterior talar process fracture was performed in this study. The AOFAS and VAS scores were used to analyze the treatment outcomes. The percutaneous screw fixation and ORIF techniques were performed for posterior talar process fracture in accordance with fracture displacements.

Considerable improvements were observed in the AOFAS and VAS scores at 4 and 12 months postoperatively in both fixation techniques during clinical assessment. There were no considerable differences in AOFAS and VAS scores between the two techniques at 4 and 12 months postoperatively. No complications such as screw loosening, nonunion, or malunion were reported and no patients complained about stiffness of the ankle and subtalar joint. Our study suggests that the two fixation techniques that were investigated are ideal methods for posterior talar process fracture with good clinical outcomes. The small fragments were fixed using Kirschner wire and 3.0 mm headless cannulated screws were used to fix the minimal displacement of fracture fragment or comminuted fracture.

The majority of posterior process fracture’s cases are associated with concomitant subtalar dislocation. Closed reduction of the subtalar dislocation was performed initially in this study. A CT scan is recommended in order to reveal the fracture type and assess the displacement of the fracture fragment^12,29^. A minimally invasive approach, which minimized the intraoperative risk of lesion of neurovascular structures, was described and preoperative individual planning with computer-assisted three-dimensional techniques for positioned percutaneous screws was recommended. Knowledge of the exact fracture fragment and safe zone of fracture fixation can help reduce the risk of joint penetration and neurovascular bundle damage. Minimal displacement of fracture fragment with percutaneous screw fixation was recommended. The technique involved placing one or two cannulated screws to stabilize the fracture with percutaneous screw fixation. The ORIF technique with posteromedial approach was a good option for comminuted fracture and supported by previous literature^19^. Mobilization and protection of the neurovascular bundle is essential when accessing the fracture site. The methodological improvement in this study was a good option depending upon the size and displacement of the fracture fragment. Findings suggest that the minimally invasive fixation of posterior talar process fracture is potentially a safer and more convenient method rather than the ORIF with posteromedial approach.

Twelve cases were treated with these two techniques with satisfying clinical results overall. There was no evident difference between the two, according to clinical assessment. Recognizing the influence of the anatomic region on the accuracy of assessing the screw position preoperatively can help the surgeon avoid placing screws that penetrate the articular facet or injuring the neurovascular bundle and tendon, as described in previous literature^8,15^. Cannulated screws are recommended for fracture fixation with small fracture fragments and articular cartridge.

## Limitations

The limitations in this study are several. Firstly, deviation may be present in the results due to the nature of the research being retrospective and most importantly due to the limited number of cases. In addition, the short follow-up time might have not been adequate for complications such as ankle and subtalar joint osteoarthritis to develop. Another limitation is the validity and reliability of the AOFAS and VAS score system, which is not universally examined and accepted. Future studies should include more clinical cases or cadaveric studies can be used to validate these techniques. A future randomized and controlled study with larger number of cases is necessary to obtain more reliable results and draw conclusions for both techniques.

## Conclusion

This study provided insight into our previous experience using percutaneous screw fixation and ORIF techniques for the treatment of posterior talar process fracture with clinical outcome assessment. Implementation of the minimally invasive technique allows easy access for fracture fixation, minimization of intraoperative risk of traction lesion of neurovascular structures, and shortening of operation time and rehabilitation period. The study provided valuable information for the minimally invasive technique of posterior talar process fracture and contributed to the accuracy of this highly demanding technique. Additionally, ORIF with posteromedial approach was recommended for fixations of fracture fragments with large displacements that may cause incongruity in the subtalar or ankle joint. The postoperative VAS and AOFAS scores were comparable, with no significant differences between the two techniques. Early diagnosis and timely determination of the displacement of the fracture are necessary for the choice between the using of percutaneous screw or ORIF technique. Screw placement can be done within the safe zone, avoiding lesion of neurovascular structures, with careful preoperative planning. Further future, randomized trials will help determine whether percutaneous screw fixation associated with posterior talar process fracture truly influences the rate of subtalar arthritis formation when compared with the ORIF technique with posteromedial approach.

## Patients and methods

### Patients

The current study was a retrospective, observational investigation, in which patients diagnosed with fractures of talar posterior processes involving an isolated fracture of either medial or lateral tubercle or entire posterior process fractures participated from January 2014 to December 2018. This study was conducted in accordance with the World Medical Association Declaration of Helsinki and approved by the Ethics Committee of the affiliated hospital of the Medical School of Ningbo University. Informed consent was obtained from the patients who participated in this study. Among the twelve patients that participated in this study, eight were male and four female, of Asian origin, with an average age of 38 years, ranging from 25 to 58 years. The cause of injury for eight of the cases was vehicle accident and for four of the cases sport injury. Seven patients exhibited posterior process fractures associated with concomitant subtalar dislocation, three talar body involvement and two concomitant avulsion fracture of lateral malleolus. The types of fractures that the patients exhibited were one lateral tubercle fracture, three medial tubercle fractures and eight entire posterior process fractures. Exclusion criteria included surgeries on open posterior process fractures and additional procedures due to talar body and neck fractures. Among a total of 12 patients have undergone pre and post-operative 3D-CT.

### Pre-operative planning

The posterior talar process fractures present in the study were classified as lateral tubercle fracture, medial tubercle fracture and entire posterior process fracture. A shift > 2 mm indicated significant displacement with surgical treatment. Internal fixation was designed to perform as internal fixation compression with a cannulated screw.

Seven cases of posterior process fractures associated with concomitant subtalar dislocation were reduced and stabilized by emergency cast. The remaining cases were cast before preoperative examination. All CT scans were performed using a 256-slice Siemens CT scan (GE, United States) with 1.0 mm slices at 0.1 s intervals for the foot and ankle imaging. The raw data were acquired in DICOM format and reconstructed into 3D models using the software MIMICS 15.01 (Materialise's interactive medical image control system, Leuven, Belgium, https://www.materialise.com/mis)^15^. Any fractures that exhibited displacement larger than 2 mm involving joint surface were treated with percutaneous screw fixation or ORIF. The neurovascular and soft tissue conditions were assessed and recorded preoperatively. Swelling was a factor to the time of surgery. ORIF was performed when soft tissue swelling was reduced, while swelling did not affect the percutaneous screw fixation procedure.

**Figure 1:**
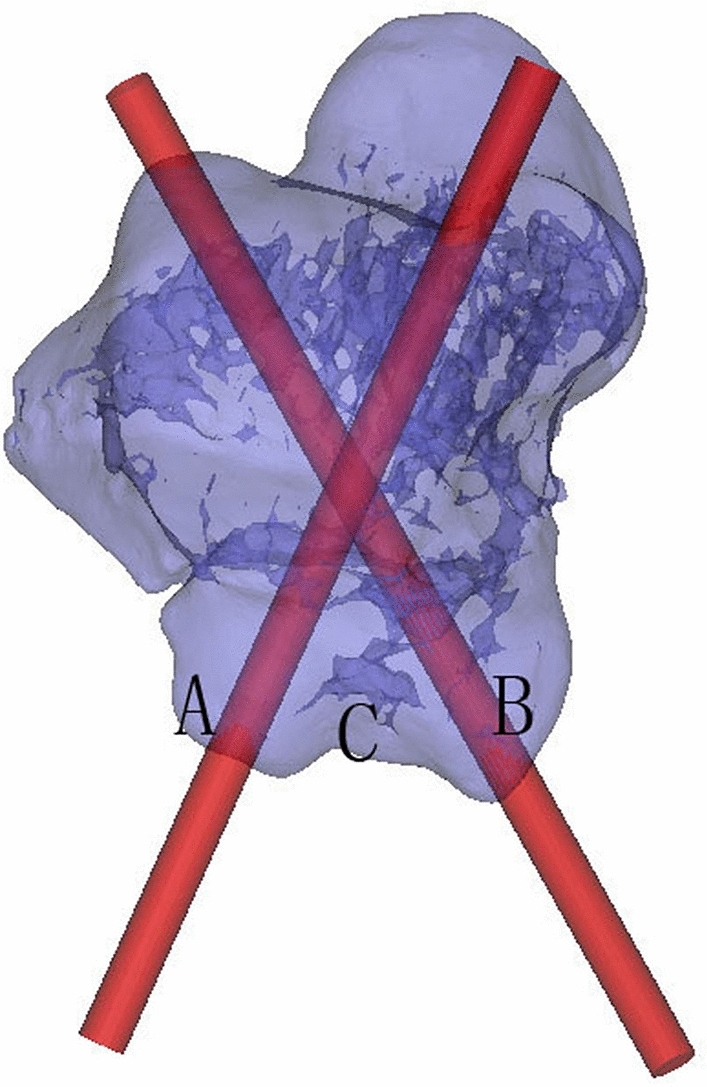
The 3D model with medial and lateral virtual screw path of the posterior talar process. (**A**) Lateral tubercle of posterior talar process; (**B**) medial tubercle of posterior talar process; (**C**) groove of the flexor hallucis longus tendon.

Simulation of inserting the virtual computer-aided design (CAD) screw (diameter 3.0 mm) was performed, after obtaining three-dimensional (3D) reconstructions of original CT scans. One or two screws were positioned for fixation of the medial and lateral tubercle of the posterior talar process (Fig. 1). The achieved position of each screw was verified using the 3D reconstructions and three different 2D images, taken in three different planes (axial, coronal and sagittal), thus enabling assessment of potential perforation into the subtalar or the ankle joint^15^. This technique offered a good evaluation of the fixation for talar process fractures preoperatively.

### Surgical technique

**Figure 2:**
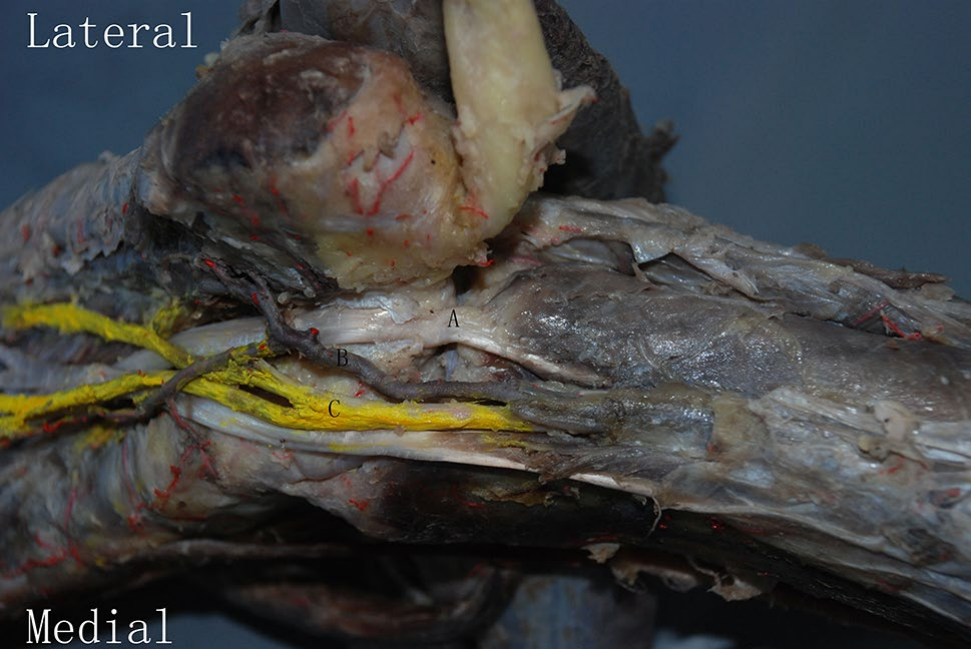
Cadaver specimen with tibial neurovascular bundle accompanied with the FHL tendon, which is located at the level of the posterior ankle joint. (**A**) Flexor hallucis longus tendon; (**B**) posterior tibial artery; (**C**) plantar nerve.

**Figure 3:**
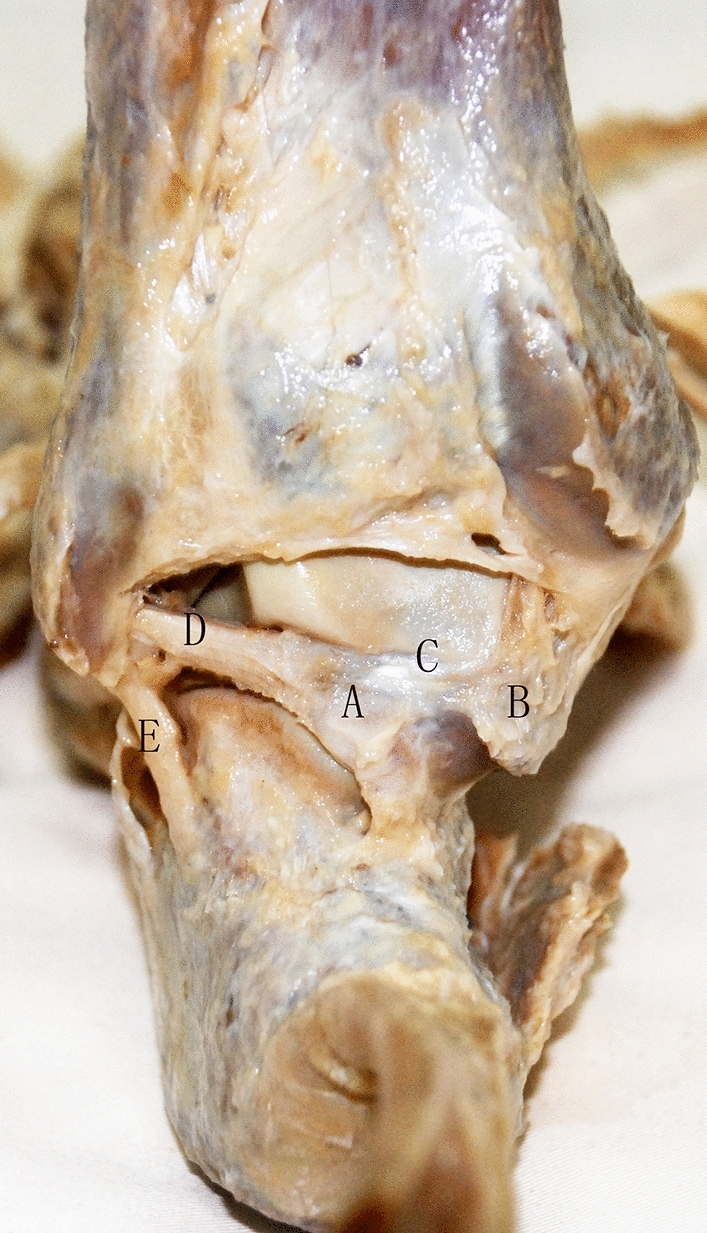
Cadaver specimen with posterior ankle joint and posterior talar process. (**A**) Lateral tubercle of posterior talar process; (**B**) medial tubercle of posterior talar process; (**C**) groove of the flexor hallucis longus tendon; (**D**) posterior talofibular ligament; (**E**) calcaneofibular ligament.

**Figure 4:**
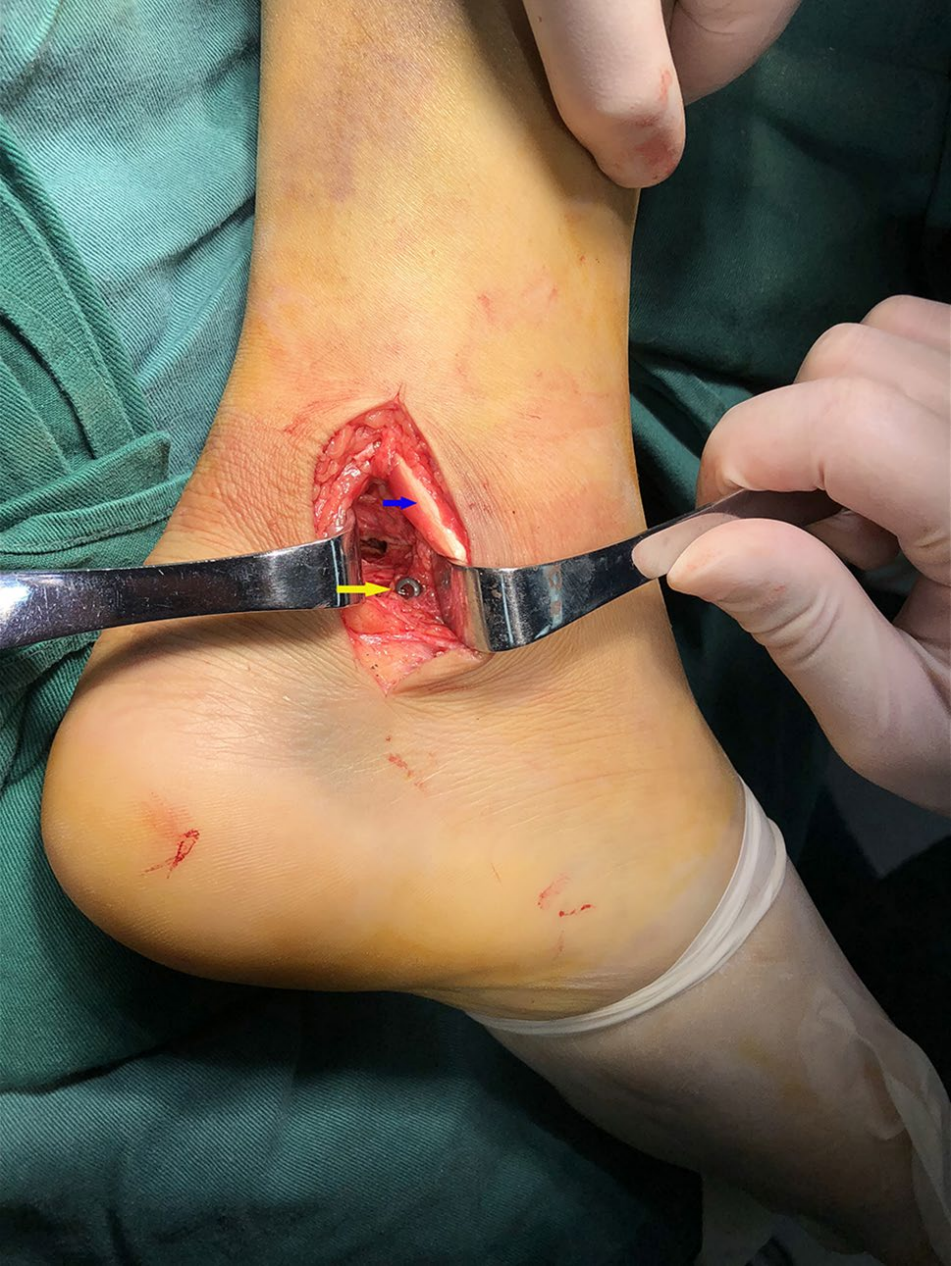
Intraoperative photo of ORIF. The yellow arrow is a cannulated screw. The blue arrow is the flexor hallucis longus tendon.

Surgery was performed under general or epidural anesthesia and the patient was placed in prone position, with a well-padded tourniquet placed at the proximal thigh. The approach of the ORIF surgical technique was modified according to the method described in Shi et al^19^. Skin incision was performed from the medial malleolus proximally to the calcaneal tuberosity distally and then the incision was continued along the middle line between the anterior edge of the Achilles tendon and the posterior border of the medial malleolus. The skin and subcutaneous tissue were carefully dissected when close to the posterior tibial neurovascular bundle (Fig. 2). Once the posterior tibial neurovascular bundle and the flexor hallucis longus tendon were identified, they were retracted medially to achieve excellent exposure of the medial tubercle, lateral tubercle and FHL tendon groove (Fig. 3). The FHL tendon served as a marker of fracture reduction. The fracture fragments were reduced and fixed temporally with Kirschner wires. One or two 3.0 mm cannulated screws were applied as a lag screw function (Fig. 4).

**Figure 5:**
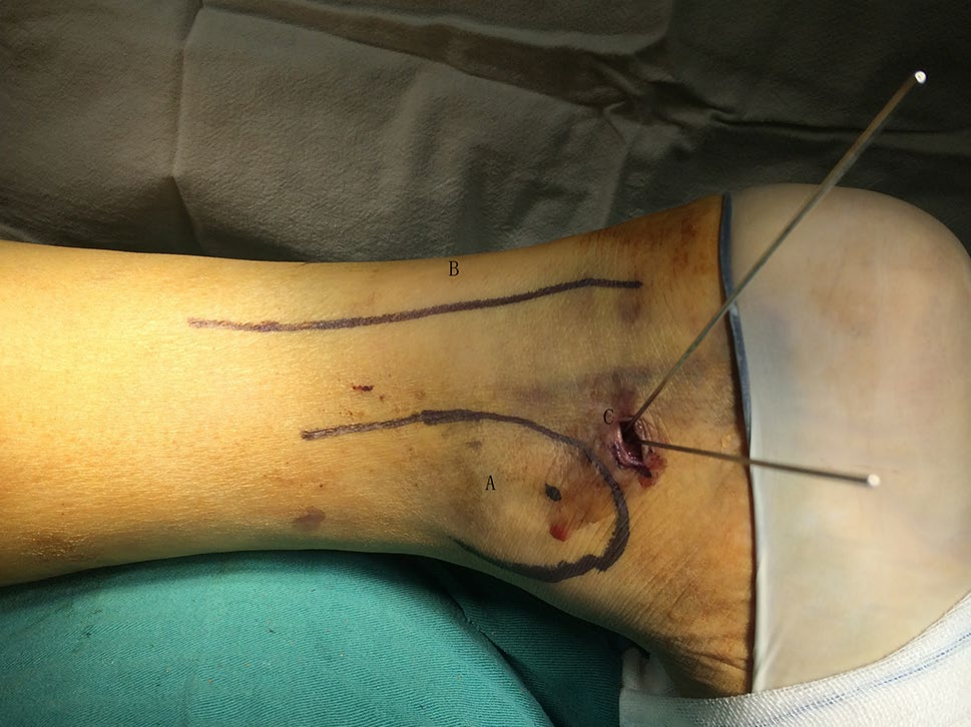
Intraoperative photo of percutaneous screw implanted with the minimally invasive technique. (**A**) Medial ankle; (**B**) achilles tendon; (**C**) entry point of percutaneous screw.

**Figure 6:**
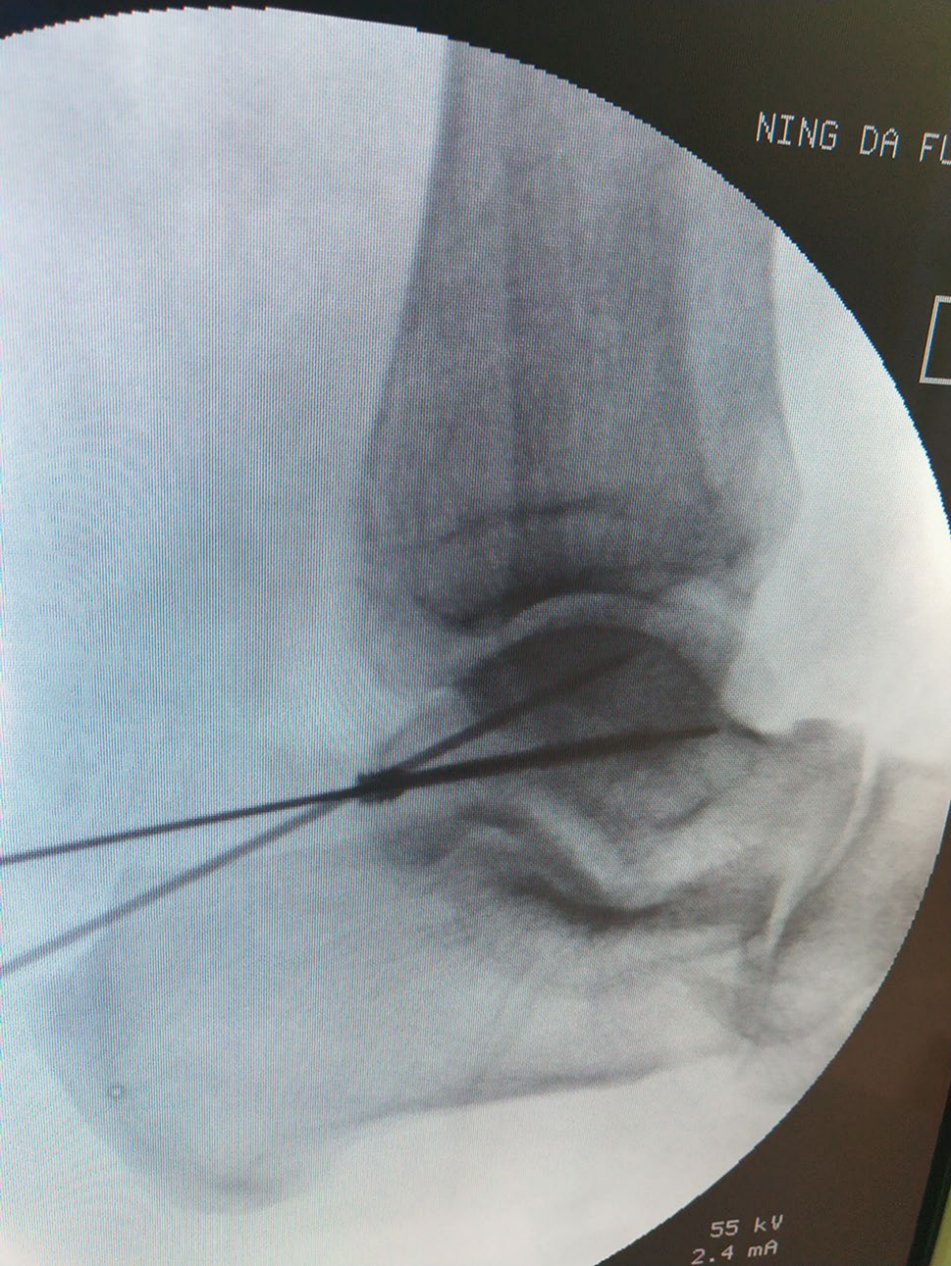
Intraoperative fluoroscopy photo of percutaneous screw implanted with the minimally invasive technique.

The minimally invasive surgical technique of fracture fixation was modified according to the method described in Mao et al.^8,15^. A closed reduction and internal fixation of the posterior process of the talus using a minimally invasive approach was performed, based on the images from the preoperational simulations (Fig. 5). The fragment was then fixed with a self-tapping and drilling 3.0 mm cannulated screw (Synthes Inc, Shanghai) directed from the posterior to the anterior through the fracture fragment into the body of the talus (Fig. 6). The fracture was then reduced temporarily and stabilized with two guide pins and the reduction was confirmed with fluoroscopy.

### Postoperative management

**Figure 7:**
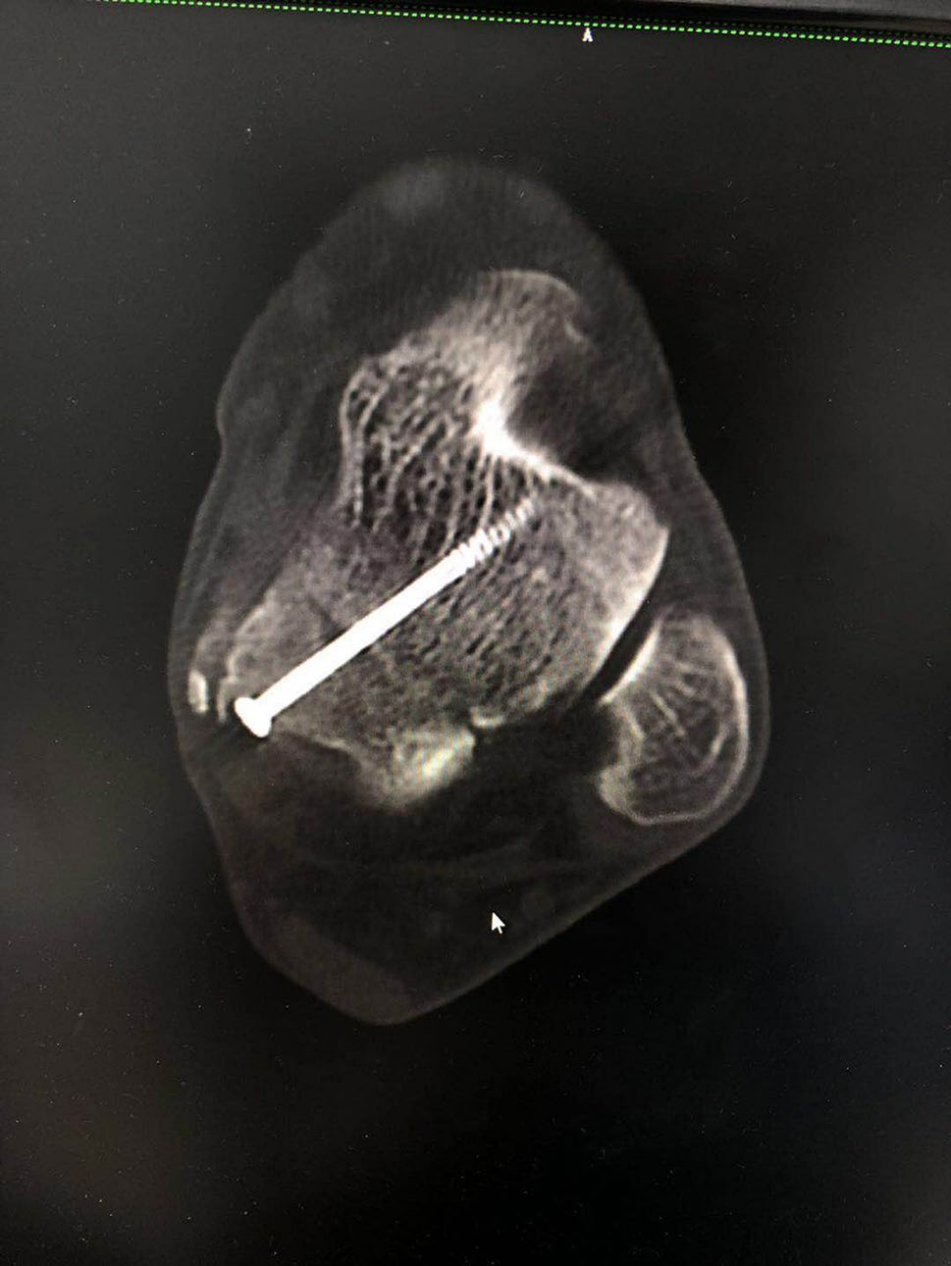
Axial view of CT scans showing that the internal fixation was performed properly.

The wound was closed and stabilized using a dry and sterile bandage, then a below-knee splint was applied, and patients were permitted non-weight bearing mobility with the use of crutches. The internal fixation of the fractures was confirmed with X-ray and CT scans after one day of operation (Fig. 7). Skin sutures were removed 14 days postoperatively and the operated limb was immobilized with a short non-weight bearing leg cast for another 6 weeks. Physical therapy consisting of passive and active assisted and resisted ankle range-of-motion exercises was performed during these days^8^.

### Clinical and radiological evaluations

All patients were treated with the same postoperative procedures, casted with below knee splints for six weeks. Clinical and radiological outcomes of patients were assessed at 4 and 12 months postoperatively. The postoperative course of the patients was evaluated based on Visual Analog Scale (VAS). Pain score and function were evaluated using the American Orthopedic Foot and Ankle Society (AOFAS) Ankle–Hindfoot score. The AOFAS scores were categorized as excellent (90–100 points), good (80–89points), fair (70–79 points), and poor (69 points)^13^. It should be noted, that the AOFAS score is currently not validated for most authors in the literature.

### Statistical analysis

Statistical analysis was performed using the Statistical Package for Social Sciences (SPSS, IBM) version 20.0. The statistical significance of the difference between the groups was determined at the 0.05 significance level^15^. The Mann Whitney U test was used for the skewed distribution of cases. Statistical differences between the times of clinical and radiographic results were analyzed using One-Way ANOVA test followed by post hoc multiple comparison with Bonferroni test.

### Ethics statement

The experiment was conducted in accordance with the Declaration of Helsinki (World Medical Association). Informed consent was obtained from the patients who participated in this study. This research was approved by the Ethics Committee of the affiliated hospital of the medical school of Ningbo University.
